# ZBP-89 function in colonic stem cells and during butyrate-induced senescence

**DOI:** 10.18632/oncotarget.21698

**Published:** 2017-10-09

**Authors:** Ramon Ocadiz-Ruiz, Amanda L. Photenhauer, Michael M. Hayes, Lin Ding, Eric R. Fearon, Juanita L. Merchant

**Affiliations:** ^1^ Department of Internal Medicine, Division of Gastroenterology, University of Michigan, Ann Arbor, MI, USA; ^2^ Division of Molecular Medicine, University of Michigan, Ann Arbor, MI, USA; ^3^ Department of Molecular and Integrative Physiology, University of Michigan, Ann Arbor, MI, USA

**Keywords:** organoids, Apc, CDKN2A, ChIP-Seq, SA-bGal

## Abstract

ZBP-89 (*Zfp148, ZNF148*) is a Kruppel-type zinc-finger family transcription factor that binds to GC-rich DNA elements. Earlier studies in cell lines demonstrated that ZBP-89 cooperates with Wnt β-catenin signaling by inducing β-catenin gene expression. Since β-catenin levels are normally highest at the crypt base, we examined whether ZBP-89 is required for stem cell maintenance. Lineage-tracing using a *Zfp148Cre*^*ERT2*^ transgenic line demonstrated expression in both intestine and colonic stem cells. Deleting the *Zfp148* locus in the colon using the *Cdx2NLSCre*^*ERT2*^ transgene, reduced the size and number of polyps formed in the *Apc*-deleted mice. Since colon polyps form in the presence of butyrate, a short chain fatty acid that suppresses cell growth, we examined the direct effect of butyrate on colon organoid survival. Butyrate induced senescence of colon organoids carrying the *Apc* deletion, only when *Zfp148* was deleted. Using quantitative PCR and chromatin immunoprecipitation, we determined that butyrate treatment of colon cell lines suppressed *ZNF148* gene expression, inducing *CDKN2a* (*p16*^*Ink4a*^) gene expression. Collectively, *Zfp148* mRNA is expressed in CBCs, and is required for stem cell maintenance and colonic transformation. Butyrate induces colonic cell senescence in part through suppression of ZBP-89 gene expression and its subsequent occupancy of the *CDKN2A* promoter.

## INTRODUCTION

Zinc Finger Binding Protein-89 kDa (ZBP-89, human *ZNF148* or mouse *Zfp148 loci*) is a ubiquitously expressed *Krüppel*-type zinc finger transcription factor that binds GC-rich DNA elements [1, 2]. We previously showed that histone deacetylase inhibition (HDACi), e.g., with butyrate or trichostatin A (TSA) contributes to ZBP-89 induction of *CDKN1B* (*p21*^*Waf1*^) and its protein-protein interaction with tumor suppressor proteins p53, ataxia telangiectasia mutated (ATM), and p300, a histone acetyltransferase (HAT) [2-4]. In addition, ZBP-89 mediates transcriptional repression of the vimentin and *CDKN2A* (*p16*^*INK4A*^) genes by recruiting HDACs to their promoters [5-7]. ZBP-89 induces *tryptophan hydroxylase* (*Tph1*) by synergizing with β-catenin, and contributes to the mucosal defense against *S. typhimurium* [8]. Having established that ZBP-89 and β-catenin cooperate in normal colonic mucosal restitution, we tested and found that ZBP-89 contributes to intestinal polyp formation initiated by deletion of the *Apc* locus [9].

Conditional deletion of the *Zfp148* locus in intestinal epithelial cells reduced the expected number of small intestinal polyps in a mouse model of deleted *Apc,* demonstrating that ZBP-89 is required for β-catenin-dependent polyp formation [9]. We found that β-catenin binds to the *ZNF148* promoter stimulating ZBP-89 gene expression. Similarly, ZBP-89 protein binds to the *CTNNB1 (*β-*catenin*) promoter inducing β-catenin transcription. In this way, ZBP-89 contributes to a feedforward gene expression loop that can maintain elevated levels of β-catenin when and where Wnt signaling is high [9], such as in the normal stem cell niche and in colon cancer. Accordingly, we also showed that ZBP-89 expression correlates with poor survival after surgical resection for colorectal cancer and that ZBP-89 protein expression is elevated in colorectal cancer (CRC) [9].

In both the intestine and colon, Wnt signaling is highest at the base of the glands or crypts suggesting that ZBP-89 might play an essential role in the stem cell niche. To test this hypothesis directly, we studied the expression and function of ZBP-89 in intestinal and colonic stem cells and found that the transcription factor is expressed in the crypt basal columnar cells (CBCs) that eventually differentiate and repopulate the small intestine and colon glands. Moreover, ZBP-89 is also required for polyp growth in the colon of hemizygous *Apc (Cdx2Cre:Apc*^*FL/+*^*)* mice. To study the effect of a common bacterial metabolite on stem cell maintenance, colonic organoids generated from wild type (WT) and *Cdx2Cre:Apc*^FL/+^ mouse colons were cultured in the presence of the short-chain fatty acid (SCFA) butyrate. We found that organoids generated from the *Cdx2Cre:Apc*^*FL/+*^ colon retain their stem cell phenotype in the presence of butyrate. However, deleting *Zfp148* decreased colonic organoid growth and allowed the organoids to senesce. Collectively, our results demonstrate that ZBP-89 plays a role in stem cell differentiation during homeostasis and transformation through its ability to suppress senescence.

## RESULTS

### ZBP-89 protein is highly expressed in CBCs and transit-amplifying (TA) cells

**Figure 1:**
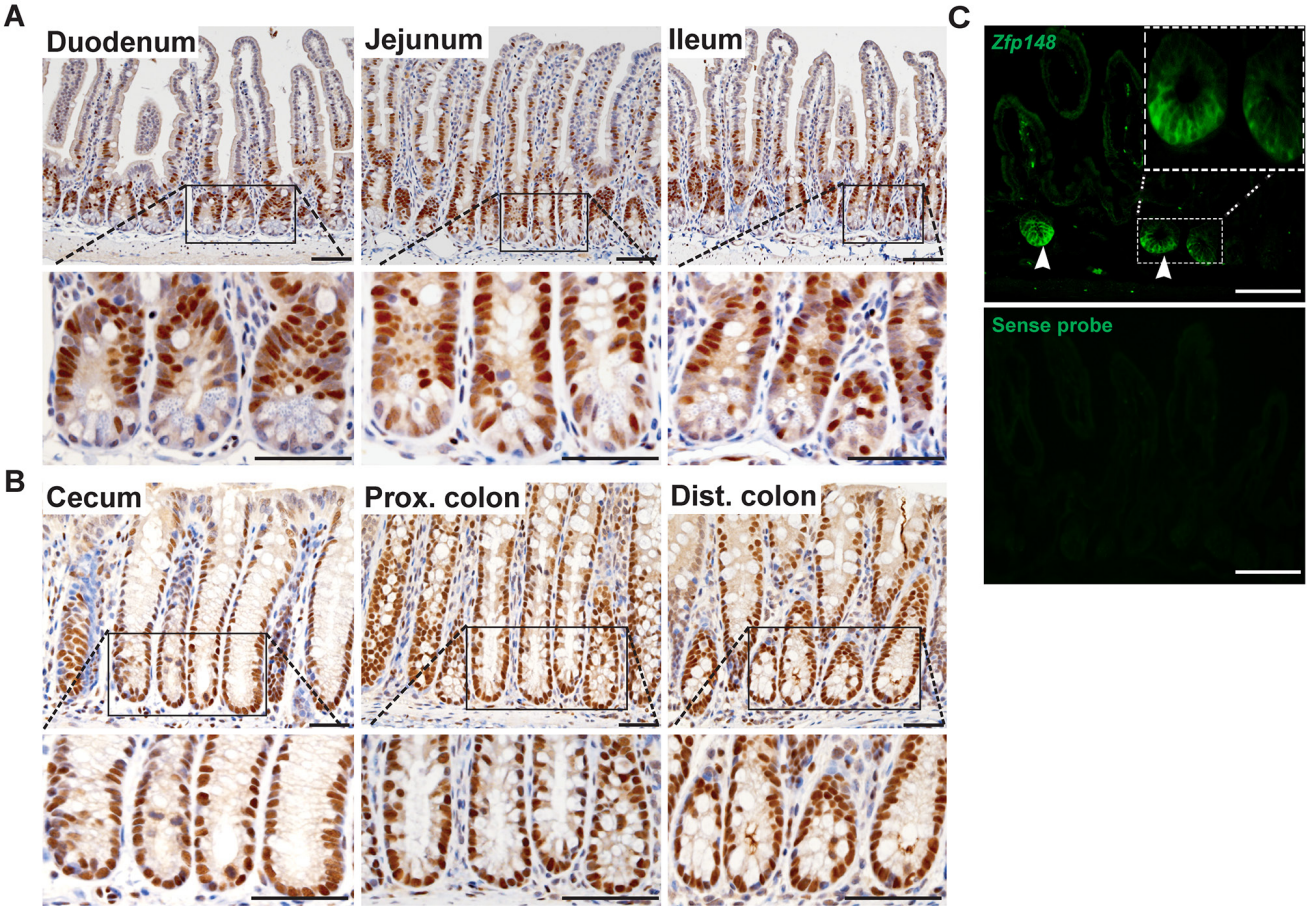
ZBP-89 is highly expressed in CBCs and TA cells Representative images of immunohistochemistry for ZBP-89 in the **(A)** small intestine and **(B)** colon. **(C)** *Zfp148 in situ* hybridization in the small intestine. The sense probe was used as the negative control. Scale bar=100μm. Inset of CBCs at 200μm.

To determine the mucosal location of ZBP-89 protein expression in the luminal gastrointestinal tract, we performed immunohistochemistry (IHC) on tissue from the small intestine (duodenum, jejunum and ileum) and colon (proximal and distal colon). In the small intestine, ZBP-89 protein was expressed primarily in the transit-amplifying (TA) cells and in scattered cells at the crypt base (Figure 1A). Expression diminished at the villus tip, where senescent cells slough into the lumen. In the cecum and colon (proximal and distal), ZBP-89 was expressed in the lower two-thirds of the colonic gland base (Figure 1B). In addition, ZBP-89 is a ubiquitous protein that is also highly expressed in immune cells [10, 11], accounting for expression in scattered cells of the lamina propria.

To localize *Zfp148* mRNA, we performed *in situ* hybridization (ISH) in the small intestine and found that the highest level of mRNA expression occurred at the crypt base (Figure 1C). Scattered cells in the lamina propria also expressed *Zfp148* mRNA, consistent with its known expression in immune cells (Figure 1) [10].

### *Zfp148+* cells lineage trace in small intestine and colon

**Figure 2:**
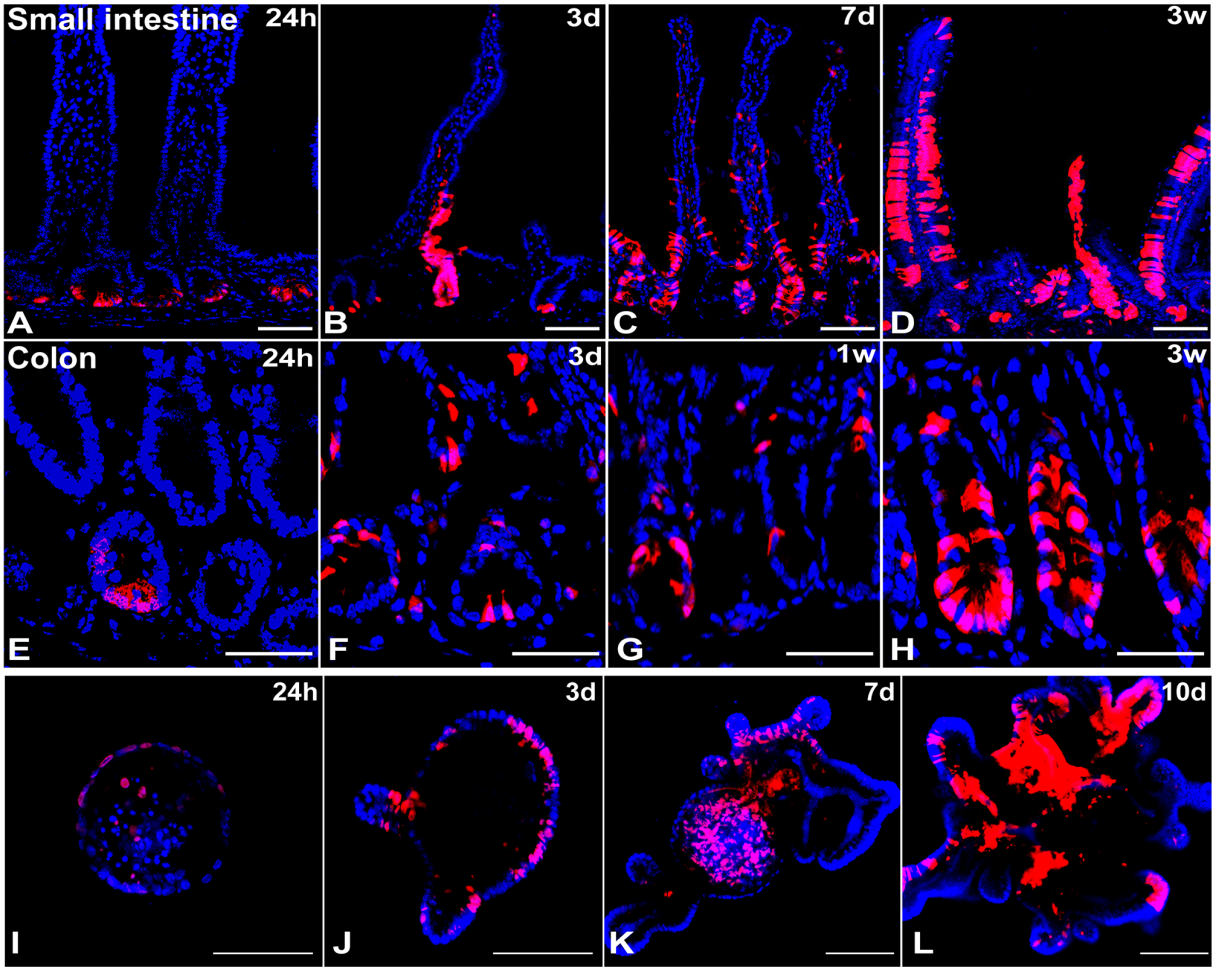
Lineage trace of *Zfp148* in mouse small intestine and colon Representative pictures of *Zfp148* lineage trace using *Zfp148: tdTomato* chimeric mice IP injected with one dose of tamoxifen 0.1mg/g body weight (Tx). The small intestine was examined after 24h **(A)**, 3 days **(B)**, one week **(C)** and three weeks **(D)**. The colon was examined after 24h **(E)**, 3 days **(F)**, one week **(G)** and three weeks **(H)**. **(I-L)** Confocal images of intestinal organoids cultured over 10 days after generating from the *Zfp148: tdTomato* mouse injected with one dose of Tamoxifen and euthanized after 24h. N=4 mice analyzed per time point. Scale bar=100 μm.

Since *Zfp148* mRNA was expressed at the base of the crypts in CBCs, we generated a *Zfp148Cre*^*ERT2*^ line from a BAC clone containing 250 kb of the *Zfp148* promoter to lineage trace its expression. The *Zfp148: tdTomato* hybrid mice were injected with one dose of tamoxifen (Tx) and then were euthanized at 24h, 3d, 1 week and 3 weeks post injection. At 24h post Tx injection, *Zfp148* positive cells resided at the base of the small intestine and colonic crypts (Figure 2A, 2E). By day 3, the intestinal crypt base and transit-amplifying (TA) cells were consistently labeled (Figure 2B, 2F). By 7d post injection, the tdTomato+ (tdT^pos^) cells populated not only the crypts and TA zone but also labeled scattered cells at the tip of the villi (Figure 2C, 2G). After 3 weeks, *Zfp148* positive cells formed stripes along the entire villus and colonic glands (Figure 2D, 2H). As previously observed for the *Lgr5-EGFP-Cre*^*ERT2*^ reporter [12], we also found that the *Zfp148Cre*^*ERT2*^ plasmid also generated a variegated lineage trace. While the variegation might indicate stem cell heterogeneity, recent modifications to the original Lgr5 reporter (*Lgr5-2A-EGFP-Cre*^ERT2^) created non-variegated lineage tracing, which suggests that the patchy expression is related to transcript stability [13]. Intestinal glands were harvested 24h post Tx injection from the chimeric *Zfp148:tdT* mice and cultured for 10 days in Matrigel to generate *Zfp148:tdT*^*pos*^ organoids. Two days after culture, a few *Zfp148:tdT*^*pos*^ cells were visible in the spherical organoids (Figure 2I). By day 3, the *Zfp148:tdT*^*pos*^ cells localized to the crypt-buds and onto the villus-like domain (Figure 2J). At day 7 and 10, *Zfp148:tdT*^*pos*^ cells were predominantly located in the crypt-like structures and in the organoid lumen (Figure 2K, 2L). Thus, we concluded that *Zfp148:tdT*^*pos*^ cells are highly expressed in the CBC stem cells and can repopulate the entire villi, indicating that ZBP-89 plays a role in both intestine and colonic stem cells.

### *Zfp148* positive cells form small intestine and colonic organoids

**Figure 3:**
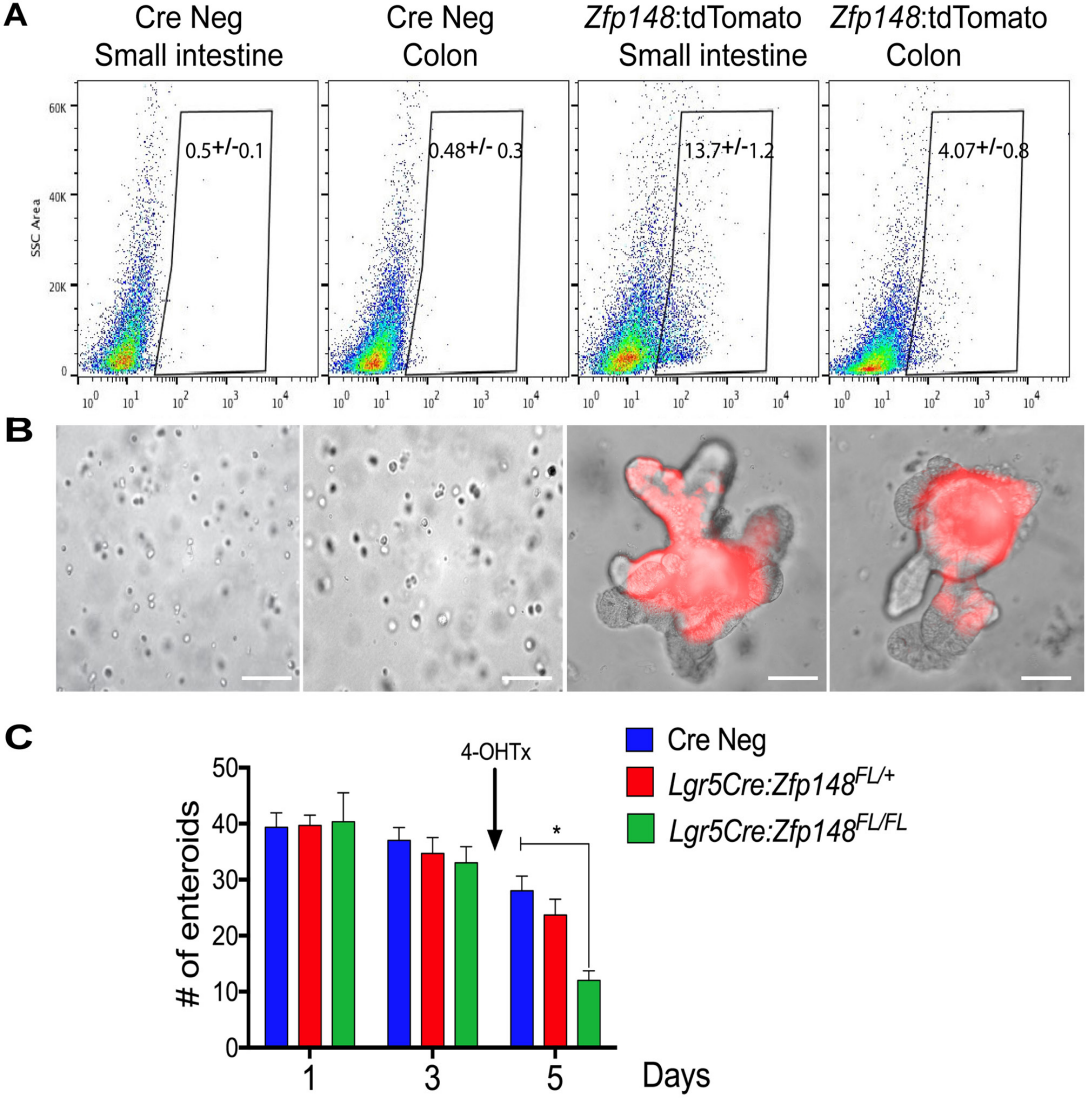
Single cell sorting of *Zfp148:* tdTomato^+^ cells and deletion of *Zfp148* in stem cells **(A)** Single cell sorting of *Zfp148: tdTomato* positive cells from the small intestine and colon. Gate of fluorescent cells isolated from the CreNeg versus *Zfp148Cre*^*ERT2*^*+* mice 24h after one dose of Tx (0.1mg/g body weight). Shown is the median percentage ±SEM for tdTomato+ sorted cells from N=3 mice per tissue per genotype. **(B)** Representative images of sorted cells cultured in Matrigel after 3 days in culture. **(C)** Graph showing total number of small intestine organoids per day. Shown is the mean ±SEM. ^***^ *P<* 0.0001 ANOVA with Tukey’s multiple comparisons. N=3 passages of organoids pooled from 3 mice and cultured in triplicate per time point. Arrow indicates the addition of 4-OH-Tx added after the organoids were in culture for 3 days. Scale bars =100μm.

To further assess the function of ZBP-89 in small intestinal and colonic stem cells, single tdT^pos^ cells were sorted from a cell suspension of small intestine or colonic mucosa 24h post Tx injection (Figure 3A). Both *Zfp148tdT*^*pos*^ and *Zfp148tdT*^*neg*^ cells isolated from the intestine and colon were re-suspended in Matrigel and cultured in complete media. Only the *Zfp148tdT*^*pos*^-sorted cells were capable of forming organoids (Figure 3B). Thus, *Zfp148-*tdT^+^ cells from both the intestine and colon generated budding organoids that continued to express the tdTomato reporter (Figure 3B). To study the function of ZBP-89 in the *Lgr5*+ CBC stem cells, the *Zfp148* locus was deleted by treating the *Lgr5Cre*^*ERT2*^*:Zfp148*^*FL/+*^ and *Lgr5Cre*^*ERT2*^*:Zfp148*^*FL/FL*^ chimeric mouse organoids with 4OH-Tx after 3 days in culture. After 48h of 4OH-Tx treatment, the number of *Lgr5Cre*^*ERT2*^*:Zfp148*^*FL/FL*^ organoids were significantly reduced compared to the Cre Negative (CreNeg) and *Lgr5Cre*^*ERT2*^*:Zfp148*^*FL/+*^ (Figure 3C). Collectively, these results demonstrated that *Zfp148* plays a critical role in the survival of both intestinal and colonic stem cells.

### *Zfp148* deletion reduces tumorigenesis in *Cdx2: Apc*^*FL/+*^ mouse colon

**Figure 4:**
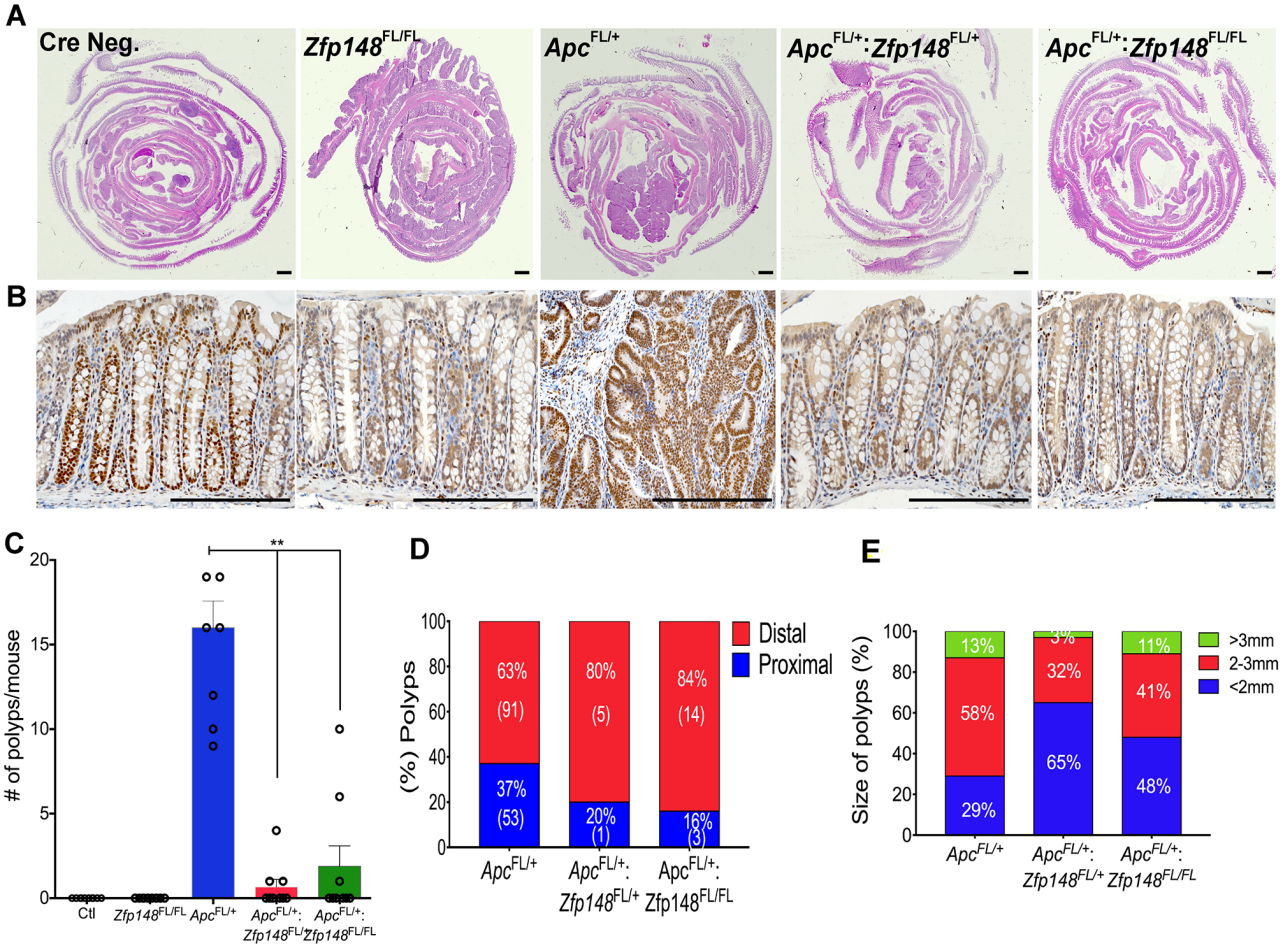
Deletion of *Zfp148* reduces colonic polyps in Cdx2: *Apc*^*FL/+*^ mice **(A)** H&E of Swiss rolls from mouse colons from Cre Negative; *Cdx2: Apc*^*FL/+*^; *Cdx2: Apc*^*FL/+*^*:Zfp148*^FL/+^ and *Cdx2: Apc*^*FL/+*^*:Zfp148*^FL/FL^ as indicated. Scale bar = 200μm. **(B)** Representative pictures of ZBP-89 IHC in the colon from the mice shown in (A). Scale bars = 200μm. **(C)** Number of polyps per mouse from the indicated genotypes. Shown are means ±SEM. N=9 mice per genotype ^**^P< 0.001, ANOVA Kruskal-Wallis test. **(D)** Distribution of polyp location as a percentage of total number of polyps per genotype. The number in parentheses equals the total number of polyps identified for that genotype for the 9 mice. **(E)** Distribution of polyp size as a percentage of total number of polyps per genotype.

To evaluate the impact of ZBP-89 on *Apc*-mediated colon polyps, we generated *Cdx2Cre*: *Apc*^*FL/+*^*: Zfp148*^*FL/FL*^ hybrid mice. The *Cdx2Cre*: *Apc*^*FL/+*^ mice [14] developed an average of 15 polyps per colon distributed throughout the proximal and distal colon after 5 months (Figure 4A-4C). The number of colon polyps in the *Cdx2Cre:Apc*^*FL/+*^ mice was ∼4-fold greater than what we observed when the *VillinCre* transgene was used to delete *Apc* [9]. In the *Cdx2Cre*: *Apc*^*FL/+*^ mice, loss of one or both *Zfp148* alleles reduced the number of colon polyps by 80% (Figure 4C). Moreover, the location of the polyps that did emerge was more distal in the *Cdx2: Apc*^*FL/+*^ mice carrying deleted *Zfp148* loci (Figure 4D) and the polyp size was significantly reduced when the *Zfp148* alleles were deleted (Figure 4E).

### *Zfp148* deletion permits colon organoid differentiation

**Figure 5:**
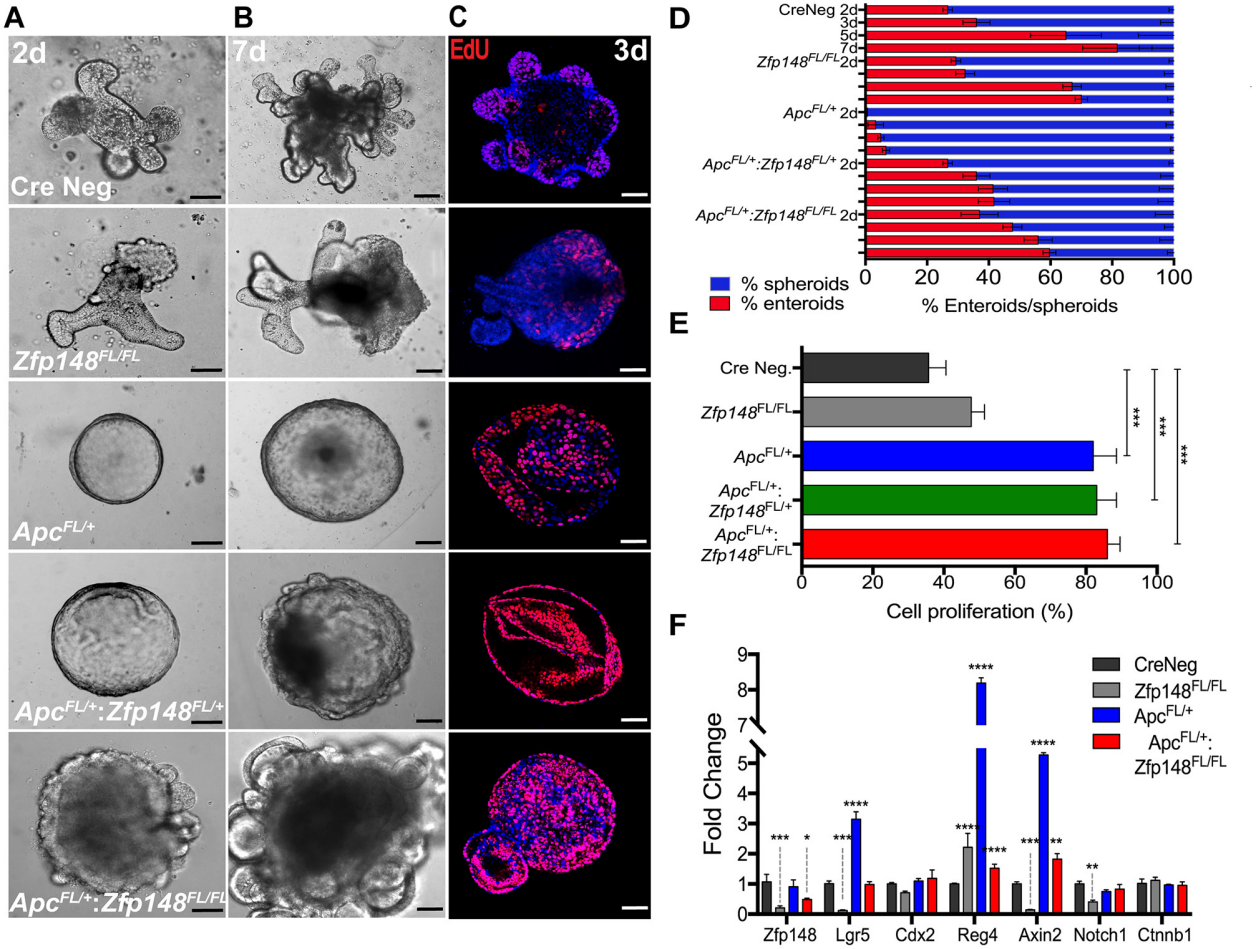
Deletion of *Zfp148* induces cell differentiation in Cdx2:*Apc*^*FL/+*^ organoids **(A, B)** Images of colonic organoid morphology for each genotype after 2 and 7 days in culture (Cre Neg.; *Cdx2: Zfp148*^FL/FL^ ; *Cdx2: Apc*^FL/+^; *Cdx2: Apc*^FL/+^:*Zfp148*^FL/+^ and *Cdx2: Apc*^FL/+^:*Zfp148*^FL/Fl^). **(C)** Confocal images of EdU incorporation per genotype after 3 days in culture. Scale bar = 100μm. **(D)** Mean percentage ±SEM for organoid morphology per total structures (organoids/spheroids) at day 2, 3, 5 and 7 after culturing in Matrigel in triplicate for three passages from 3 mice per genotype. **(E)** Mean percentage ±SEM of EdU+ nuclei, using 2-3 organoids per image. Five images per genotype per condition were quantified. **(F)** qPCR using three passages of organoids per genotype. Shown are means ±SEM, ^****^P< 0.0001 ^***^P< 0.001, ^**^P< 0.005, ^*^P< 0.05 by two-way ANOVA Tukey’s multiple comparisons test.

To directly test the effect of deleting *Zfp148* in the hemizygous *Apc* colonic stem cells, organoids were generated from the colon of *Cdx2Cre*: *Apc*^*FL/+*^ mice, which showed a non-budding stem cell morphology (spherical organoids, Figure 5A). However, loss of one or both *Zfp148* alleles was sufficient to induce a wrinkled appearance after 3 days of culture that progressed to small buds by day 7 (Figure 5A, 5B, 5D). When both *Zfp148* alleles were deleted, the organoids exhibited extensive budding demonstrating that ZBP-89 was required to maintain stemness. Although loss of one or both *Zfp148* alleles in the setting of deleted *Apc,* reduced polyp formation, it did not affect the degree of organoid proliferation as determined by EdU incorporation (Figure 5C, 5E). Although ZBP-89 was required for maintenance of the stem cell niche and cooperates with Wnt signaling, the lack of an effect on cell proliferation suggested that synergy with Wnt signaling affected a different requirement for stem cell maintenance. Therefore, we examined several Wnt targets and found that deletion of *Zfp148* in the colon decreased the expression of two direct Wnt target genes *Lgr5* and *Axin2* consistent synergy between ZBP-89 and β-catenin. Although *Reg4* a Wnt target gene was modulated by deletion of *Zfp148*, it is also regulated by Cdx2 and Gata 6 transcription factors [15-17]. β-catenin mRNA levels were not affected, perhaps reflecting steady state levels of the protein that are achieved once the organoids become established. Deleting *Zfp148* also reduced *Notch1*, suggesting some crosstalk with the Notch signaling pathway (Figure 5F).

### ZBP-89 prevents *Apc*-deleted organoids from butyrate-induced growth inhibition

**Figure 6:**
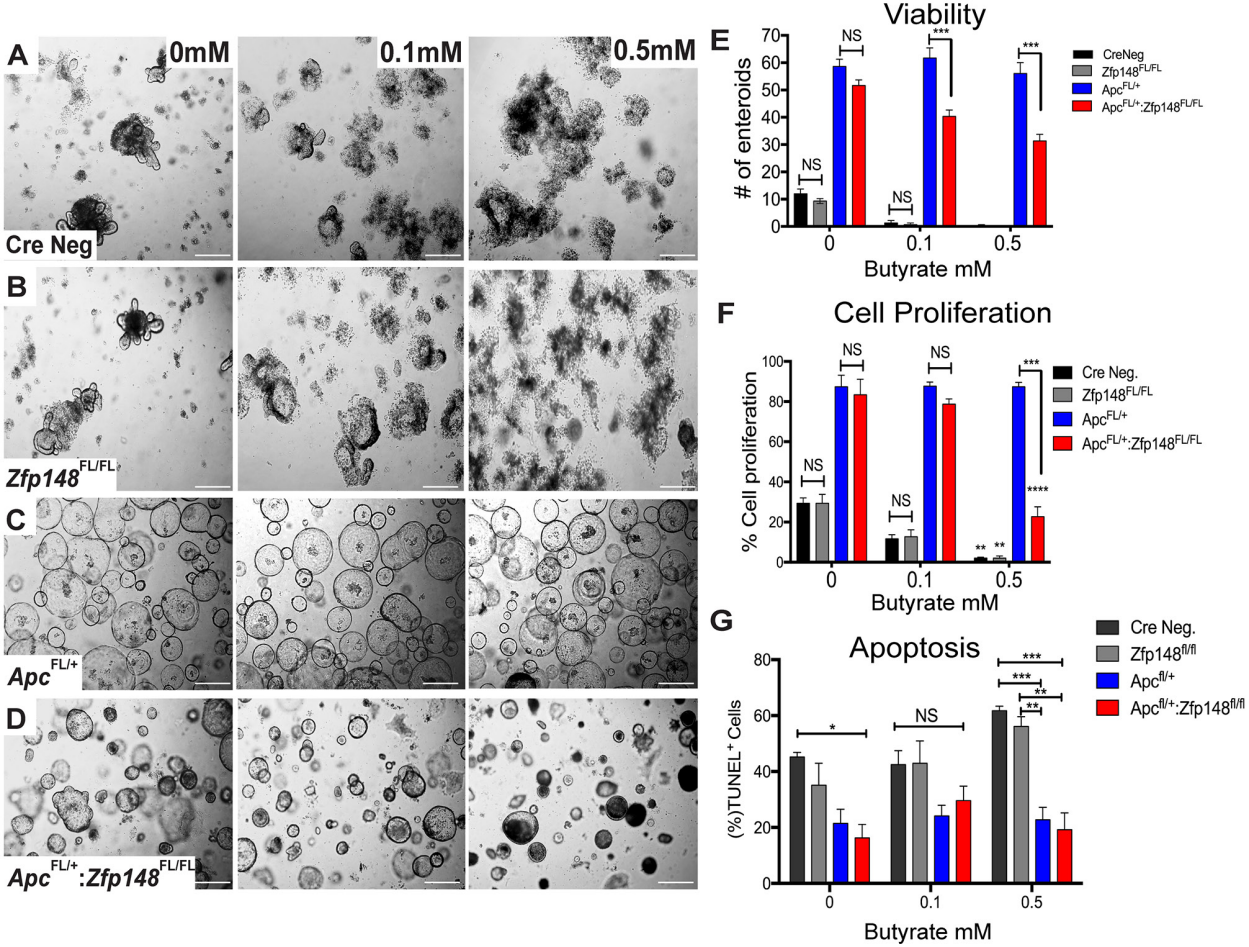
ZBP-89 protects *Apc* mutant organoids against butyrate treatment **(A, B)** Representative pictures of Cre Neg; *Cdx2:Zfp148*^FL/FL^ colonic organoid morphology after 0.1mM and 0.5mM butyrate added to the organoid culture medium **(C, D)** *Cdx2: Apc*^FL/+^:*Zfp148*^FL/+^ and *Cdx2: Apc*^FL/+^:*Zfp148*^FL/Fl^ organoids after butyrate treatment. **(E)** Number of organoids per genotype and per butyrate concentration for three passages of organoids cultured in triplicate per genotype. Shown are the means ±SEM. ^***^P= 0.001 using two-way ANOVA with Sidak’s multiple comparisons test. **(F)** Mean percentage ±SEM of EdU+ nuclei from 2-3 colon organoids per image from a pool of 3 mice per genotype. ^**^P< 0.001 and ^***^P< 0.0001 by two-way ANOVA Tukey’s multiple comparisons test. **(G)** TUNEL assay, shown as the percentage of TdT positive cells for each population per number of DAPI positive cells. ^*^P< 0.05, ^**^P< 0.001 and ^***^P< 0.0001 by two-way ANOVA Tukey’s multiple comparisons test.

**Figure 7:**
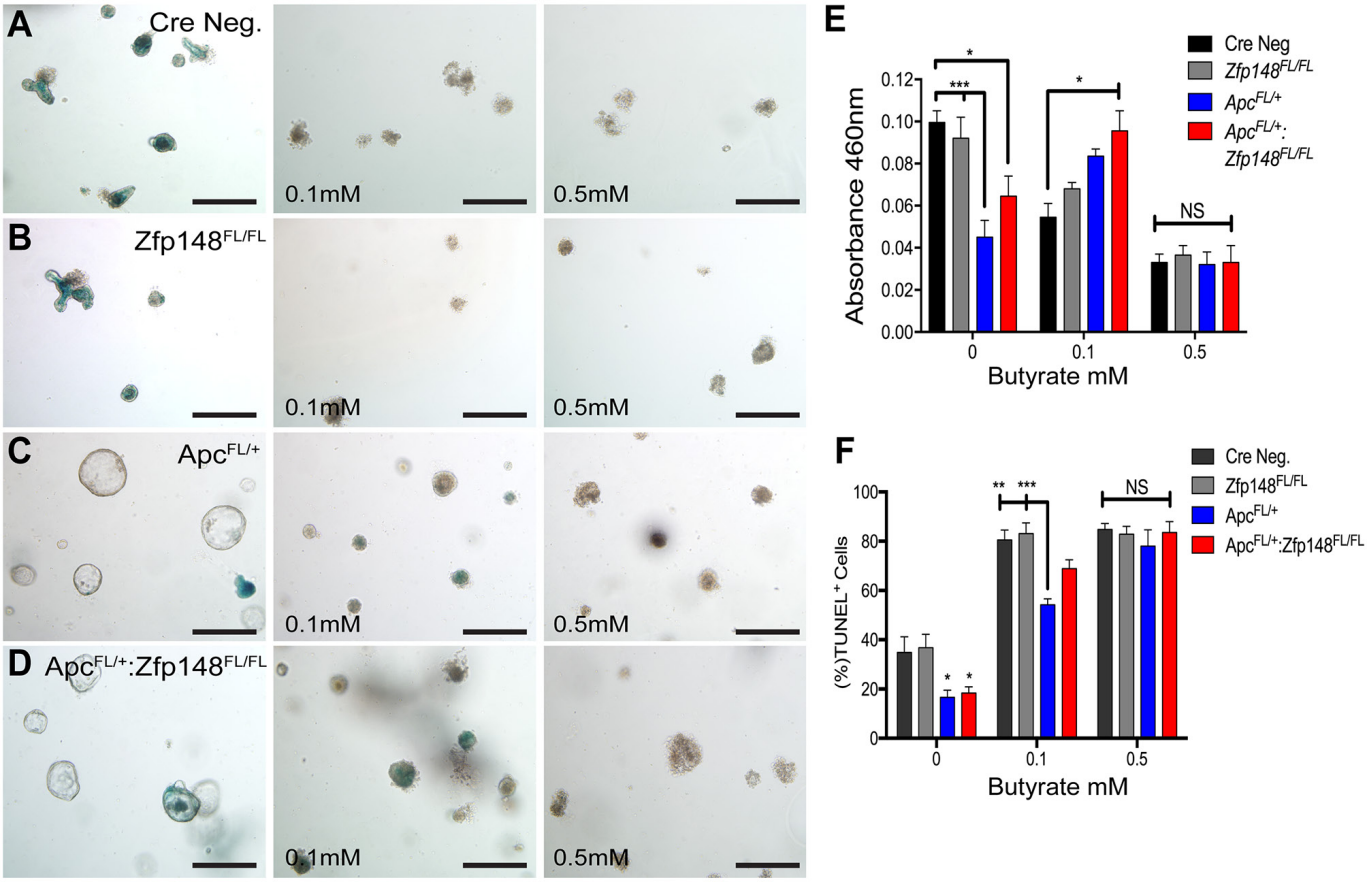
Intestinal organoids show greater sensitivity to butyrate than colon organoids Representative pictures of intestinal organoids stained for senescence-associated beta galactosidase (SA-βGal) activity before and after treating with increasing butyrate concentrations. **(A)** Cre Neg., **(B)** *VillinCre:Zfp148*^*FL/FL*^, **(C)** *VillinCre:Apc*^*FL/+*^ and **(D)** *VillinCre:Apc*^*FL/+*^*: Zfp148*^*FL/FL*^. Scale bar=100μm. **(E)** Spectrophotometric quantitation of intestinal organoids SA-βGal activity at A_460_. **(F)** TUNEL assay, shown as the percentage of TdT positive cells for each population per number of DAPI positive cells. ^*^P< 0.05, ^**^P< 0.001 and ^***^P< 0.0001 by two-way ANOVA Tukey’s multiple comparisons test.

Unlike the small intestine, tumors in the colon emerge in the presence of commensal bacteria that ferment carbohydrates into millimolar amounts of butyrate, a short chain fatty acid (SCFA) with the most potent HDAC inhibitory activity [18, 19]. Organoid cultures are usually carried out in the absence of butyrate or bacteria. Indeed, butyrate generally suppresses colonic cell growth, which might explain the persistent proliferation of the organoids carrying a mutant *Apc* allele (Figure 5). Therefore, we examined whether adding butyrate to the organoid cultures affected their growth. Colonic organoids from both WT (CreNeg) and *Cdx2Cre:Zfp148*^*FL/FL*^ mice were treated with 0.5mM butyrate, which induced a significant increase in apoptotic cells and suppressed cell proliferation compared to the group with no butyrate (Figure 6A, 6B). Under these conditions, colonic organoids carrying the *Apc* deletion (*Cdx2Cre: Apc*^*FL/+*^*)* maintained their spheroid shape and thrived in the presence of butyrate (0.5mM) even though the same concentrations of butyrate were poorly tolerated by normal colon organoids (Figure 6C, 6D). However, deletion of *Zfp148* (*Cdx2Cre: Apc*^*FL/+*^*: Zfp148*^*FL/FL*^) reduced organoid proliferation in the presence of butyrate (Figure 6E, 6F), but there was no significant increase in apoptosis (Figure 6G), suggesting that loss of *Zfp148* in the presence of butyrate might initiate cellular senescence (growth arrest) in contrast to apoptosis [20]. By contrast, intestinal organoids exhibited greater sensitivity to butyrate (Figure 7A-7E). Senescence was observed in the *Apc* and *Apc* plus *Zfp148*-deleted intestinal organoids even at 0.1 mM butyrate, while both the Cre Neg and *Zfp148*-deleted organoids without the *Apc* deletion were already apoptotic at this lower butyrate concentration (Figure 7F).

**Figure 8:**
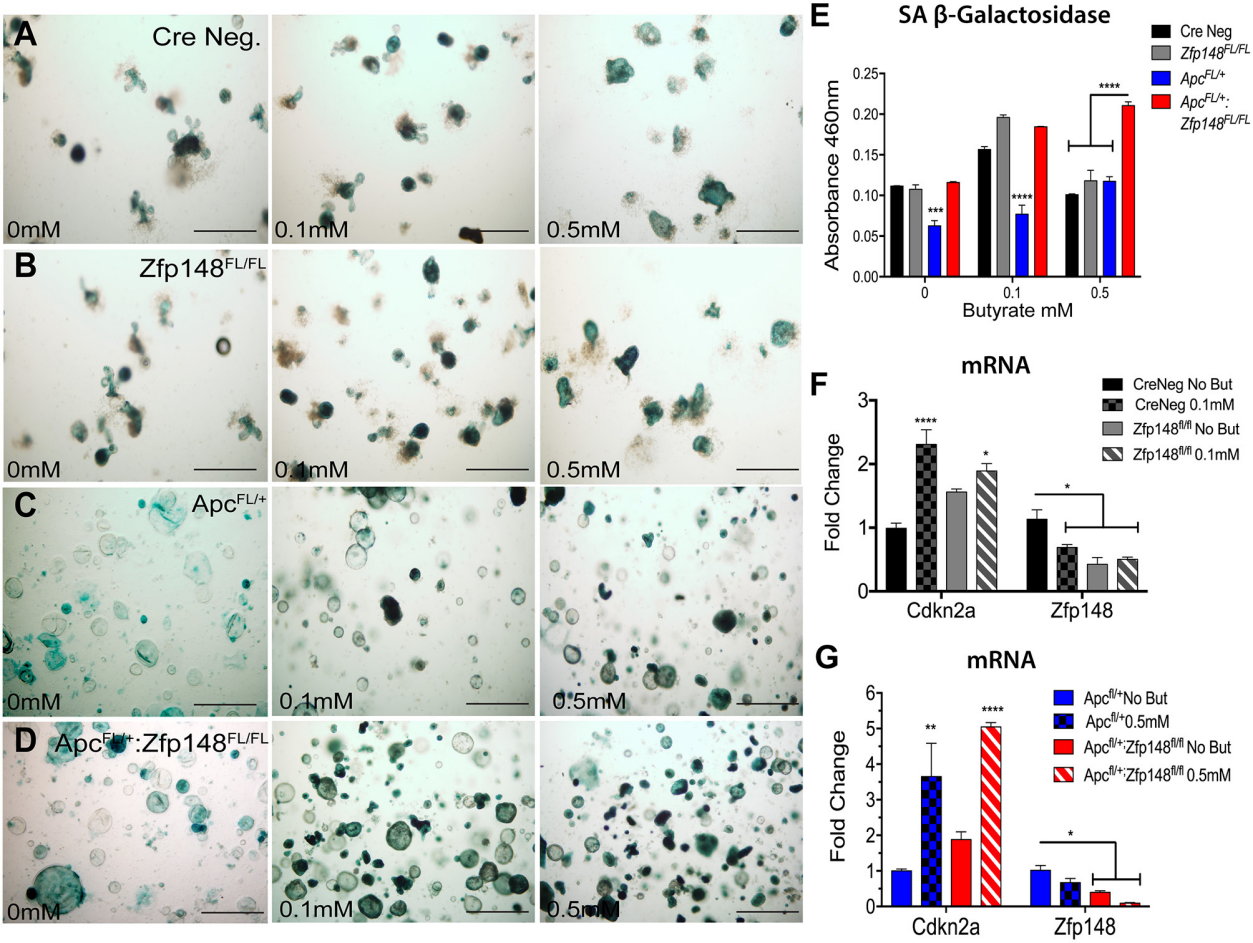
Loss of *Zfp148* in the presence of butyrate initiates cellular senescence in *Apc*^*FL/+*^ organoids Representative pictures of colonic organoids stained for senescence-associated beta galactosidase (SA-βGal) activity before and after treating with increasing butyrate concentrations **(A)** Cre Neg., **(B)** *Cdx2Cre:Zfp148*^*FL/FL*^, **(C)** *Cdx2Cre:Apc*^*FL/+*^ and **(D)** *Cdx2Cre:Apc*^*FL/+*^*: Zfp148*^*FL/FL*^. Scale bar=100μm. **(E)** Spectrophotometric quantification of colonic organoids SA-βGal activity at A_460_. **(F)** qPCR for *Cdkn2a* and *Zfp148* using three passages of CreNeg and *Cdx2: Zfp148*^FL/FL^ colonic organoids incubated with 0.1mM butyrate. **(G)** qPCR for *Cdkn2a* and *Zfp148* using three passages of *Cdx2: Apc*^FL/+^:*Zfp148*^FL/+^ and *Cdx2: Apc*^FL/+^:*Zfp148*^FL/Fl^ colonic organoids incubated with 0.5mM butyrate. ^*^P< 0.05, ^**^P< 0.001 and ^***^P< 0.0001 by two-way ANOVA Tukey’s multiple comparisons test.

To examine whether ZBP-89 colonic organoids exhibited cellular senescence, we determined the levels of SA- βGal in organoid cultures treated with increasing amounts of butyrate (0, 0.1, 0.5mM) (Figure 8). We found that both CreNeg and *Cdx2Cre: Zfp148*^*FL/FL*^ organoids were senescent and subsequently apoptotic with increasing concentrations of butyrate (Figure 8A, 8B, 8E). By contrast, *Cdx2CreApc*^*FL/+*^-derived organoids did not undergo senescence unless *Zfp148* was deleted (Figure 8C-8E). Since senescence is mediated by the cyclin-dependent inhibitor *Cdkn2a* [20], we determined its expression levels using RNA extracted from the organoids before and after butyrate treatment. Indeed, we found a significant increase in *Cdkn2a* mRNA with deletion of *Zfp148* but more dramatically when the organoids were treated with butyrate (Figure 8F, 8G). Surprisingly, butyrate strongly suppressed *Zfp148* gene expression contributing to the increase in *Cdkn2a* (Figure 8F, 8G).

**Figure 9:**
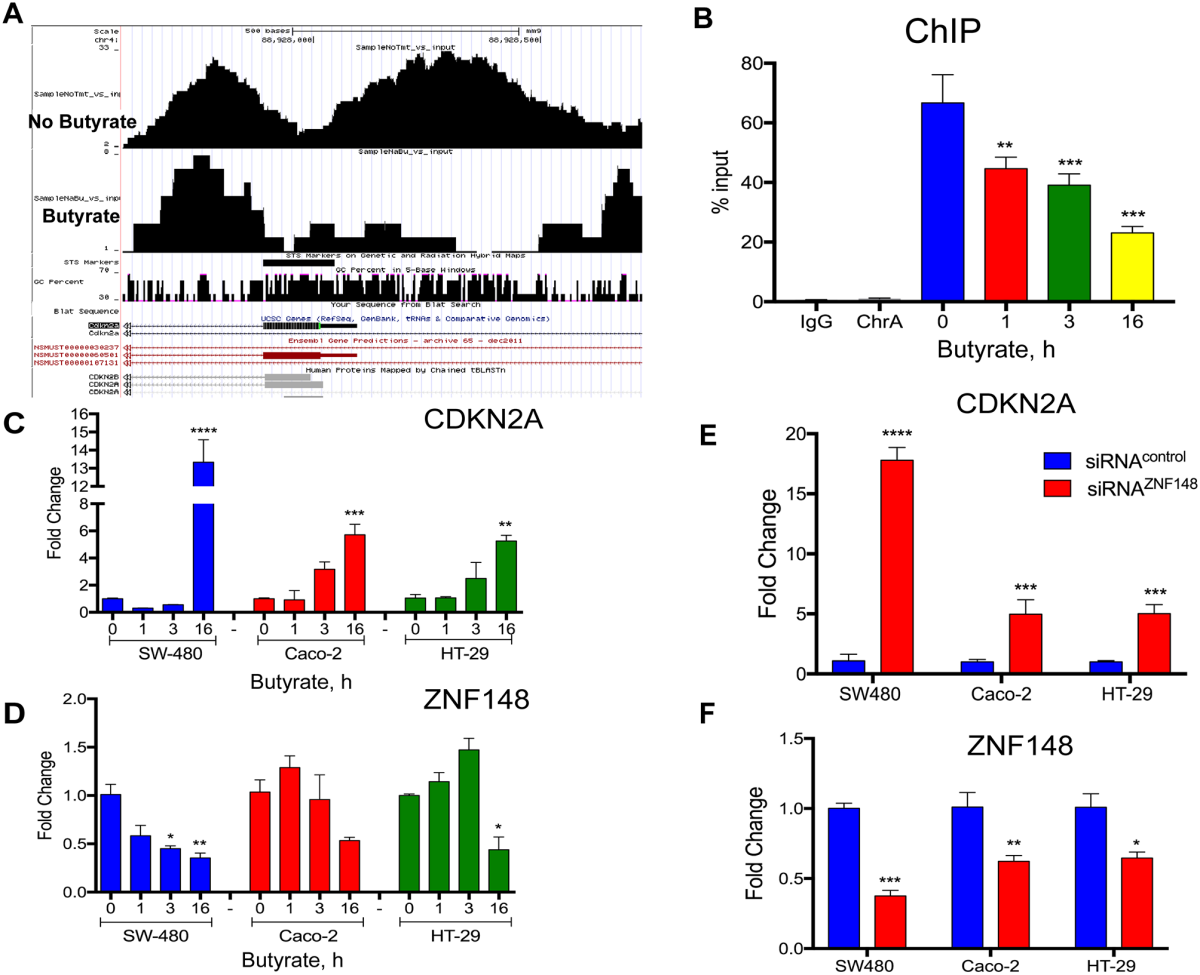
Butyrate decreased ZBP-89 binding to the mouse and human *p16*^*Ink4a*^ promoter **(A)** ChIP-Seq of mouse STC-1 cells treated with 2.5mM butyrate for 6h. Chromatin precipitated with ZBP-89 antibody was analyzed in the UCSC genome GRC m38/mm10 browser. **(B)** Chromatin Immunoprecipitation of SW480 cells treated with butyrate for 1, 3 and 16h. Occupancy of the *CDKN2A* promoter by ZBP-89 determined by qPCR was plotted as a function of the input. **(C)** SW480, Caco-2 and HT-29 cells treated with butyrate for 1, 3 and 16h were analyzed by qPCR for *CDKN2A* mRNA and **(D)** *ZNF148* mRNA. **(E)** SW480, Caco-2 and HT-29 cells incubated for 24h with *ZNF148* siRNA or siRNA scrambled control oligos and then treated with butyrate for 1, 3 and 16h were analyzed by qPCR for *CDKN2A* mRNA and **(F)** *ZNF148* mRNA. Shown are means ±SEM, ^****^P< 0.0001, ^***^P< 0.001, ^**^P< 0.05, by one-way ANOVA multiple comparisons test.

Feng et al. previously showed that ZBP-89 binds directly to and represses the *CDKN2A* promoter in a lung cancer cell line by recruiting HDACs [6]. Therefore, to determine whether butyrate regulated ZBP-89 binding to the *Cdkn2a* promoter, we performed Chip-Seq using STC-1 cells, a mouse intestinal cell line and found that ZBP-89 binding to the mouse *Cdkn2a* promoter decreases (Figure 9A). To determine if butyrate suppression of ZBP-89 binding induced *CDKN2A* gene expression and senescence in colon cancer cell lines, the CRC cell line SW480 was treated with butyrate for 1, 3 and 16h. ChiP-qPCR showed that butyrate reduced ZBP-89 binding to the *CDKN2A* promoter (Figure 9B), which corresponded to an increase in *CDKN2A* gene expression in SW480 in addition to Caco-2 and HT-29 cell lines (Figure 9C, 9D). To determine if the increase in *CDKN2A* (*p16*^*INK4A*^*)* expression corresponded directly to silencing of ZBP-89 gene expression in the colonic cell lines (SW480, Caco-2 and HT-29), *ZNF148* was knocked down using siRNA oligos. Indeed, *CDKN2A* was significantly induced and corresponded to reduced *ZNF148* mRNA expression (Figure 9E, 9F). Therefore, as observed in our mouse model, butyrate induces *CDKN2A* gene expression and presumably cellular senescence through its ability to suppress *ZNF148* gene expression.

## DISCUSSION

The studies reported here demonstrate that ZBP-89 is expressed in CBCs and plays a role in stem cell maintenance and cellular differentiation. We recently reported that ZBP-89 synergizes with Wnt signaling by directly inducing β-catenin gene expression [9]. Consequently, the increase in ZBP-89-mediated gene expression sustains elevated levels of this protooncogene. Conversely β-catenin induces the expression of *ZNF148* [9]. As previously suggested [9], the feedforward regulation of these two transcription factors contributes significantly to Wnt-driven transformation from both intestine and colonic stem cells [21]. Specifically, we show here that fewer and smaller polyps formed when both the *Zfp148* and *Apc* loci were conditionally deleted using the colon-specific *Cdx2Cre* transgene. *Zfp148* deletion corresponded to increased organoid budding indicating loss of stemness that presumably retards transformation of the colonic stem cell niche. Surprisingly, neither *Zfp148* deletion nor butyrate alone was sufficient to inhibit organoid proliferation in the context of a deleted *Apc* locus, but instead synergistically functioned to suppress the stem cell phenotype.

HDAC inhibitors such as butyrate induce cellular senescence by increasing cyclin-dependent inhibitors, e.g., *p21*^*waf1*^*, p16*^*INK4*^ [22-24]. In the colon, butyrate levels are lowest in the distal colon and deep within the crypts away from luminal bacteria [18, 19]. We previously showed that ZBP- 89 directly binds the *p21*^*waf1*^promoter and potentiates butyrate induction of this cyclin-dependent inhibitor, which is also a gene target of several transcriptional regulators including p53 and cMyc [2]. Although ZBP-89 forms a protein-protein interaction with p53 and prevents its nuclear export and degradation in the cytoplasm [3], the *in vivo* significance of the interaction was not readily apparent. A possible mechanic was through the induction p53 regulation of *p21*^*waf1*^, and subsequently cellular senescence. Recently, Lindahl and co-workers reported that ZBP-89 blocks *Apc* mutant polyp development in the intestine by inhibiting p53-mediated activity [25]. They observed no effect on cell proliferation or apoptosis in the early intestinal tumors, and suggested that reduced tumor initiation was due to a change in cell fate. Here we demonstrate that butyrate coupled with deletion of *Zfp148* was required to significantly enter a senescent phase of slowed cell growth, resulting in fewer and smaller colon polyps *in vivo* as well as fewer and smaller colonic organoids. As such, ZBP-89 has been shown to induce cellular senescence when overexpressed in lung cancer cells due to its ability to recruit HDACs to the cyclin-dependent inhibitor *p16*^*INK4A*^ promoter [6]. Thus, a contribution of ZBP-89 to the stem cell phenotype is likely related to its ability to recruit HDACs to specific promoters regulating the expression of genes that inhibit the cell cycle.

Colonic fermentation of dietary carbohydrates is the exclusive purview of commensal bacteria with butyrate being the most frequently studied of the abundant SCFAs [18]. Butyrate exhibits a number of functions ranging from colonocyte fuel source to chromatin modulation through inhibition of HDACs [26, 27]. Yet, therapy with this SCFA to suppress inflammation and transformation has been mixed, perhaps as a result of the different cellular pathways it regulates [28-30]. Moreover, cellular regulation by butyrate depends on the metabolic and proliferative status of the cell [31-33]. Typically, butyrate levels are lowest in the distal colon due to rapid fermentation of dietary fiber and use as a primary carbon source by colonocytes in the proximal colon. This in turn correlates with the propensity of colorectal tumors to develop in the distal colon where butyrate levels are the lowest [18, 32]. Indeed, the few polyps that developed in the mice carrying deleted *Zfp148* alleles were in the distal colon. In addition, butyrate levels at the base of the colonic crypt where the stem cells reside are estimated to be in the micromolar range, significantly lower than the millimolar levels at the luminal surface [32]. Consequently, we found that treating normal colonic organoids with millimolar amounts of butyrate induced senescence and subsequently apoptosis. However, organoids from *Apc*-deleted colons in which Wnt signaling is elevated protected against butyrate-mediated apoptosis and were observed to proliferate. By contrast, *Zfp148* deletion abolished the protective effect of the *Apc* deletion. Like butyrate, Zimberlin et al. showed that intestine specific deletion of both HDAC1 and HDAC2 *in vivo* or chemical ablation with a class I-specific HDAC inhibitor MS-275 reduced proliferation, expression of stem cell markers and decreased the clonogenic capacity of intestinal organoids [34]. Thus, HDACs are an essential factor in stem cell maintenance and explains in part how ZBP-89 contributes to the stem cell phenotype in the colon. We conclude that in both the small intestine and colon, ZBP-89 contributes to stem cell maintenance as a modulator of the Wnt pathway, but also through recruitment of HDACs. However intestinal organoids were more sensitive to the senescent and apoptotic effects of butyrate than those prepared from the colon. Thus, in the colon, where a gradient of butyrate is present as a function of the microbiota, we would predict that a ZBP-89-HDAC complex would inhibit *CDKN2A* and block growth inhibition as a function of being exposed to this potent natural HDAC inhibitor.

In summary, ZBP-89 plays an essential role in maintaining stemness in both the intestine and colon. As a known mediator of butyrate-dependent regulation in cell lines [2, 4], it appears that the ability of ZBP-89 to interact with HDACs contributes to its ability to synergize with β-catenin to resist the senescent effects of butyrate in *Apc* mutant stem cells where Wnt signaling and β-catenin protein levels are high. Interestingly, there appears to be tissue specific differences in the ability of the small intestine versus the colon to resist butyrate-induced senescence. Taken together, these results provide at least a partial explanation for why ZBP-89 protein expression contributes to the formation of colonic adenomas and progression during the early stages colon cancer [9, 35].

## MATERIALS AND METHODS

### Animal models

Generation of *Zfp148*^*FL/FL*^ mice on a C57BL/6 genetic background was previously described [8]. *Apc*^*FL/+*^*, Zfp148*^*FL/+*^ and *Zfp148*^*FL/FL*^ mice were bred to the *Cdx2Cre* mouse line [14] or VillinCre [36] to generate mice that were heterozygous for the *Apc* allele alone or with the *Zfp148*^*FL/+*^ and *Zfp148*^*FL/FL*^ genotypes (*Cdx2Cre*: *Apc*^*FL/+*^*:Zfp148*^*FL/FL*^*or Apc*^*FL/+*^*:Zfp148*^*FL/+*^ or VillinCre: Apc^FL/+^:Zfp148^FL/FL^ [9]). To generate the *Zfp148Cre*^*ERT2*^ transgene, the *Cre*^*ERT2*^*_IRES_eGFP* cDNA cassette was inserted downstream of the mouse *Zfp148* ATG in exon 4 prepared from BAC clone RP23-207B1 that contains 250 kb of the *Zfp148* mouse gene (JrGang Cheng, University of North Carolina). The purified *Zfp148Cre*^*ERT2*^ transgene was microinjected into the pronuclei of fertilized C57BL/6 x SJL chimeric mouse eggs (University of Michigan Transgenic Core). Three founder lines (725, 730 and 740) were bred to the *Rosa26-LoxSTOPLox-tdTomato* reporter mice (*Rosa-tdT*) (B6.Cg-*Gt(ROSA)26Sor*^*tm27.1*(CAG-COP4*H134R/tdTomatoHze^/J strain 012567) purchased from Jackson Laboratory. The lines were ultimately backcrossed to C57BL/6 mice over two years. To lineage trace *Zfp148* expression in the intestine and colon, the line with the highest fluorescent reporter expression was selected (line 730). Mice were maintained under the University of Michigan Animal Care and Use Committee, which maintains an American Association of Assessment and Accreditation of Laboratory Animal Care facility and approved all methods and procedures used.

### Immunohistochemistry

Immunohistochemistry was performed using the diaminobenzidine (DAB)-based staining technique (Abcam, ab4238). After de-paraffinization and rehydration, antigen retrieval was performed by boiling tissue sections in a microwave for 10min with 10mM sodium citrate buffer (pH 6.0). Tissue sections were preincubated with 0.3% hydrogen peroxide and nonspecific binding sites were blocked with 20% normal goat serum (Jackson ImmunoResearch, West Grove. PA). A polyclonal antibody to the conserved C- terminal residues (611-794) of ZBP-89 (1:1000, sc-48811; Santa Cruz Biotechnology Inc., Santa Cruz, CA) was incubated for 2h in a humidified chamber and then incubated with horseradish peroxidase (HRP)- conjugated secondary antibody for 30min. The sections were stained with DAB and counterstained with Mayer hematoxylin, prior to mounting in Toluene Permount (Fisher Scientific, Waltham, MA).

### *In situ* hybridization

The tissue was embedded in O.C.T. Compound (Tissue –Tek), sectioned, placed on slides and then was re-hydrated in DEPC-treated water prior to a 1h pre-hybridization incubation in 50% formamide/Denhardt’s solution at 55°C. The single strand DNA probes (ssDNA) labeled at the 5’ end with 6-FAM were reconstituted in sterile RNAse-free water, diluted to a working concentration of 50ng/ml with hybridization buffer and incubated for 48h at 60°C. Slides were stringently washed with 50% formamide in 5X sodium citrate saline (SSC, pH 7.0) buffer for 2min at 40°C. Samples were rinsed with DEPC-treated water and allowed to air-dry prior to mounting with Prolong Gold Antifade reagent with DAPI (ThermoFisher; Scientific). The following probes were used:

*Zfp148* antisense probe 1: 5’6-FAM/ACCGACT ATTAGTCCAAAGTGGGACTT/

*Zfp148* sense probe: 5’6-FAM/TGGCTGATAA TCAGGTTTCACCCTGAA/

Sense strand sequence for *Zfp148* was used as negative control.

### Mouse organoid generation

Intestinal or colonic mucosa was minced into 5mm pieces on ice followed by gland dissociation in 2mM EDTA/PBS at 4°C for 30min for intestinal tissue and 4mM EDTA/PBS at 4°C for 60min for colonic tissue. After filtration through a 70μm cell strainer (BD Bioscience) for intestine and a 100μm cell strainer (BD Bioscience) for colon, the suspension was centrifuged at 200×*g* for 5min. Intestinal glands were re-suspended in 50μl of Matrigel supplemented with Advanced DMEM/F12 (Invitrogen) containing 50% of Wnt, R-Spondin, Noggin conditioned media generated from the L-WRN (cell line ATCC® CRL-3276™) with B27 and N2 1X supplements (ThermoFisher; Scientific) plus EGF (50ng/ml, Invitrogen). To generate colon organoids, the media was mixed with Matrigel containing CHIR99021 (10mM, STEMCELL), Y-27632 (10mM, STEMCELL), A83-01 (500nM, Sigma-Aldrich) and gastrin (10nM, Sigma-Aldrich). The organoid preps were cultured at 37°C in 5% CO_2_. Butyrate treatment of intestinal or colonic organoids was performed 3 days after the organoids became established in Advanced DMEM/F12 media. Upon receipt, frozen stocks were generated for the L-WRN cells and were checked for mycoplasma every 3 months. EdU staining of colonic organoids was performed for 16h using the Click-iT® EdU Imaging Kit and was developed according to manufacturer’s instructions. EdU incorporation was quantified using the program Cellprofiler (http://cellprofiler.org/) comparing EdU area to DAPI stained nuclei per image [37]. Five images per passage of organoids derived from 3 mice per genotype per condition were analyzed.

### *In vitro* tamoxifen induction

To activate the Cre^ERT2^ transgene in culture, the organoids were incubated with low dose 4-hydroxytamoxifen (4-OH-Tx, 100nM) for 24h in complete Advanced DMEM/F12, (Invitrogen). After the incubation, the cultures were washed with PBS and then fresh complete media was added.

### Real-time quantitative PCR

Total RNA from intestine and colon was extracted and purified, using the PureLink RNA Mini Kit (ThermoFisher). RNA (1μg) was DNAse-treated (Promega) and used to generate cDNA using the iScript reverse transcriptase kit (Bio-Rad, Hercules, CA). Real-time quantitative polymerase chain reaction (RT-qPCR) was performed using Platinum Taq DNA polymerase (Invitrogen) on a CFX96 real-time PCR detection system (Bio-RAD), using the following primer sequences (Tm = 65°C for all primers):

*Zfp148*: F: 5’ TCCAAACCACTGATTCTTCTCTT; R: 5’ AGTTCTCTCCCCTCCCCCT

*Lgr5*: F: 5’ TCTTCTAGGAAGCAGAGGCG; R: 5’ CAACCTCAGCGTCTTCACCT

*Cdkn2:* F: GCAGAAGAGCTGCTACGTGA; R: 5’ CGTGAACATGTTGTTGAGGC

*Cdx2*: F: 5’ TCTGTGTACACCACCCGGTA; R: 5’ GAAACCTGTGCGAGTGGATG

*Reg4*: F: 5’ GCACAGCTGGGTCTCAAGAT; R: 5’ CATCGAAAGAGGAAGATGGC

*Axin2*: F: 5’ TGCATCTCTCTCTGGAGCTG; R: 5’ ACTGACCGACGATTCCATGT

*Notch1*: F: 5’ CTGAGGCAAGGATTGGAGTC; R: 5’ GAATGGAGGTAGGTGCGAAG

*Ctnnb1*: F: 5’ CAGCTTGAGTAGCCATTGTCC; R: 5’ GAGCCGTCAGTGCAGGAG.

### Flow cytometry and cell sorting

Isolated crypts were incubated in culture medium for 45min at 37°C, followed by cell dissociation using Accutase (STEMCELL) for 15min at 37°C. Dissociated cells were filtered through a 40μm pore cell strainer (BD Bioscience). TdTomato ^positive^ cells were sorted by flow cytometry using an iCyt Synergy Flow sorter (Sony Biotechnology). Single viable epithelial cells from both the intestine and colon were gated by forward scatter, side scatter, pulse-width parameter and by negative staining for DAPI (Sigma Aldrich). Sorted cells were collected in crypt culture medium, embedded in Matrigel and then overlaid with complete culture media.

### TUNEL assay

Three-day old small intestine or colonic organoids were resuspended in cold cell dissociation with 2mM EDTA/PBS and incubated at 4°C for 10min, and then filtered through a 40μm cell strainer (BD Bioscience). The suspension was centrifuged at 200×*g* for 5 min, pelleted and then resuspended in 1ml of 3.7% formaldehyde. After incubating for 10 min, the fixed cells were washed in cold PBS and labeled using the TACS 2 TdT fluorescein kit according to manufacturer’s procedure (Trevigen, MD). The total number of TdT positive cells for each population was calculated using gating percentage multiplied by total number of cells labeled with DAPI (Sigma Aldrich). Analysis was performed using FlowJo software (TreeStar, Ashland OR).

### Senescence-associated galactosidase activity assay

Galactosidase activity was quantified in intestinal and colonic organoids using the Senescence Cells Histochemical Staining kit (Sigma Aldrich) according to manufacturer’s instructions. Staining was developed over 10h and visualized in an inverted optical microscope (Leica). For quantitation, intestinal or colonic organoids were lysed in RIPA buffer (ThermoFisher) for 10min and centrifuged at 10,000×*g* for 5min, the supernatant was collected and the absorbance was measured on a plate reader at A_460._

### Chromatin immunoprecipitation (ChIP)

SW480 cells were treated with 1% formaldehyde and then quenched with glycine for 5min at room temperature. Cell lysates were sonicated to shear DNA into fragments of 200 to 1000bp (8 cycles of 30 second intervals) using a 130W Sonic Vibracell (VCX130PB). An aliquot (5%) of the lysates was removed and used as the Input, while the remaining solution was used to immunoprecipitate cross-linked protein using a ZBP-89 monoclonal antibody and an IgG antibody as control. Immune complexes were captured using Protein A/G agarose beads, after washing, bound proteins were eluted from the beads and diluted 1:15 in deionized water (EZ Magna ChIP A/G kit, Millipore). After proteinase K and RNase A digestions, purified DNA was analyzed by PCR using NovaTap DNA polymerase (Millipore) using flanking promoter primers for *CDKN2A* and primers for chromogranin A (ChrA), a gene not regulated by ZBP-89. ChIP-qPCR values were expressed as a percentage of Input DNA.

### ChIP-seq

Mouse STC-1 cells were treated with 2.5 mM butyrate for 6h before adding 1% formaldehyde. After performing chromatin immunoprecipitation (ChIP) with ZBP-89 antibody (Santa Cruz, CA), generation of the library and Illumina sequencing was performed by the UM Sequencing Core. Conversion of the sequences into Fastq was performed using Illumina’s CASAVA-1.8.2 software. Q-Scores were determined using ASCII+33. Genomic signatures were analyzed for GC-rich ZBP-89 consensus sites within 1kb of the transcriptional start sites (TSS). Duplicate sequencing reads per treatment were aligned to the Mouse GRC m38/mm10 and BAM files were converted to BigWig for analysis in the UCSC genome browser.

### Statistical analysis

Two-way ANOVA with Tukey’s multiple comparisons test was performed to analyze the number of organoids and cell proliferation assays. One-way ANOVA with Kruskal-Wallis multiple comparisons test was used to compare the number of polyps per genotype. Two-way ANOVA with Sidak’s multiple comparisons test was used to compare the size of organoids per genotype per condition.

## Abbreviations

SCFA: short chain fatty acids
tdT: tdTomato
Tx: tamoxifen
HDAC: histone deacetylase
